# Tactile sensitivity in the rat: a correlation between receptor structure and function

**DOI:** 10.1007/s00221-021-06193-7

**Published:** 2021-09-14

**Authors:** Lucia Guzun, Pascal Fortier-Poisson, Jean-Sébastien Langlais, Allan M. Smith

**Affiliations:** Centre de Recherche en Sciences Neurologiques, Département de Physiologie, Université de Montréal, C.P. 6128 succersale centre-ville, Montréal, QC H3C 3J7 Canada

**Keywords:** Cutaneous receptors, Meissner corpuscles, Merkel complexes, Rapidly adapting, Slowly adapting

## Abstract

Single cutaneous fibers were recorded in the median nerve of the deeply anesthetized rat and the receptor morphology in the forelimb glabrous skin was analyzed to establish a probable correlation between receptor anatomy and physiology. Receptor complexes in the glabrous skin of the rat forelimb were stained immunologically with antibodies NF-200 and PGP-9.5, confirming the presence of Meissner corpuscles and Merkel complexes within the dermal papilla similar to other mammals including primates. Both the Meissner corpuscles and Merkel cell complexes were sparse and located in the pyramidal-shaped palmer pads and the apex of the digit extremities. They were almost totally absent elsewhere in the glabrous skin. No Ruffini receptors or Pacinian corpuscles were found in our samples. A total of 92 cutaneous fibers were retained long enough for analysis. Thirty-five (38%) were characterized as rapidly adapting fibers (RA) and 57 (62%) were slowly adapting afferents (SA). Despite the very limited number of receptors at the tip of the digit, RA receptors outnumbered SA fibers 3.2/1.0. In contrast, SA fibers on the thenar pad outnumbered RA receptors by a ratio of 3–1. Despite the very limited number of low threshold mechanoreceptors in the glabrous skin of the rat forelimb, the prevalence of SA afferents in the palm and more frequent occurrence of RA afferents in the digit extremity suggest differences in functionality both for locomotion and object manipulation.

## Introduction

The relationship between specialized skin receptors and the sensation of touch has been of interest to anatomists, physiologists and psychologists for well over a century. In spite of the early speculative enthusiasm of the nineteenth century anatomists, Weddell and Miller (1962), in their review of cutaneous sensibility, offered the opinion that given the current state of the art, it was impossible to provide a definitive account of the relationship between skin receptor morphology and receptor physiology. No doubt this assertion, along with improved anatomical and electrophysiological techniques, incited a number of investigators to address this challenge in subsequent studies (Brown and Iggo 1967; Iggo and Muir 1969; Iggo and Ogawa 1977; Janig 1971; Parduz et al. 1977). Similarly, Mountcastle and collaborators (Talbot et al. 1968; Werner and Mountcastle 1965) addressed the relationship between receptor physiology and behavioral response thresholds using behavioral psychophysics. Nevertheless, the only receptor for which an unequivocal connection between function and structure has been demonstrated is the Pacinian corpuscle (RA II); a receptor so large and of such low density that it could be dissected and studied electrophysiologically. This enabled a clear physiological demonstration of its sensitivity to high frequency vibrations over a substantial distance, and its rapidly adapting property (Bell et al. 1994; Lowenstein and Mendelson 1965). A second type of slowly adapting afferent in the hand (RAII) has been shown to respond to lateral skin stretch in particular directions (Johansson 1978) and, although it has been suggested that the morphological substrate of this response might be the Ruffini ending, this assertion has been seriously questioned (Paré et al. (2002).

Despite considerable research with three different research strategies, the morphological identity of rapidly adapting type I and slowly adapting type I receptors remains controversial. The most commonly employed method used to establish the identity of these receptors was to record from an isolated skin afferent, precisely label the receptive field on the skin with lamp black and then to examine the underlying tissue histologically (Iggo and Muir 1969; Iggo and Ogawa 1977; Janig 1971; Munger et al. 1971; Parducz et al. 1977; Saxod 1978). The immediate consensus was that the SAI receptors were associated with Merkel endings in the hairy skin of the cat. This assertion was supported by Janig (1971) who examined the hairless skin of the cat hind paw but warned that the identity of 6/13 SAI receptors was uncertain. However, the very high density of Merkel endings and Meissner corpuscles particularly in the monkey hand created an uncertainty about the respective correlations with rapidly and slowly adapting receptor activity.

A second method of investigation consisted of comparing electrophysiological recordings before and after the application of neurotoxic agents. Ikeda et al. (1994) injected quinacrine, a substance with a strong affinity for Merkel cells, into the skin of the rat. In theory, when exposed to blue light (420–490 nm) the quinacrine fluoresces and destroys the Merkel cell. After 20 min of blue light exposure, Ikeda et al. (1994) reported SA activity was abolished and subsequent analysis demonstrated Merkel cell degeneration again supporting the general consensus. Still, these results were challenged by Mills and Diamond (1995) who reported that the slow adaptation of 33/39 receptors was virtually unaffected by the destruction of the majority of Merkel cells. Furthermore, a similar study by Senok et al. (1996) indicated that the quinacrine toxicity of Merkel cells was neither selective nor complete.

A third line of research used transgenic mice to alter skin innervation, again with somewhat equivocal results. For example, Carroll et al. (1998) used brain-derived neurotrophic (BDNF)-deficient mice to demonstrate a significant reduction in slowly adapting mechanical responses to sustained stimulation without a corresponding decrease in the number of Merkel cells or significant change their morphology. Kinkelin et al. (1999) also found that neurotrophin p7.5-deficient mice had a 99% reduction in Merkel cells without any change to the threshold or the properties of the SA responses. In contrast, Airaksinen et al. (1996) using NT-3 knockout mice produced a 76% reduction in Merkel cell count and a corresponding loss of the large fiber slowly adapting mechanical response strongly supporting the association of Merkel cells with the SAI response pattern.

A fourth line of research has addressed the problem directly by labeling physiologically identified skin sensory neurons in the neonatal mouse (Woodbury et al. 2001; Woodbury and Koerber 2007). The spinal cord and thoracic dorsal root ganglia (DRG) along with the dorsal cutaneous nerves and dorsal trunk skin were dissected and maintained with an oxygenated medium. DRG cell were then impaled with quartz recording electrodes and subsequently labelled with Neurotbiotin. Electrophysiological recordings identified the receptors as belonging to the SAI category as they exhibited typical slowly adapting sustained discharges in response to light punctate contact applied to their receptive fields.. The short distance involved allowed Neurotbiotin to diffuse into the peripheral processes as well as into the postganglionic terminal dendritic arborization in the dorsal horn. The histological examination showed that even in neonates the terminal were adult-like expanded disc-like endings on Merkel cells in the basal epidermis.

Finally, two recent studies using genetically modified mice have produced somewhat puzzling results. Maricich et al. (2012) produced mice devoid of Merkel cells and found that these mice were unable to detect textured surfaces with their feet while other measures of motor and sensory function were unaffected. Neubarth et al. (2020) produced a BDNF deficient mouse that was devoid of Meissner corpuscles and their innervating sensory neurons, although the density of Merkel complex appeared to be normal. Electrophysiological recording found no RAI responses in the hind limb glabrous skin despite the presence of SAI responses. In contrast to, Maricich et al. (2012), these BDNF deficient mice showed significant deficits in skin indentation thresholds and in object manipulation dexterity.

The rat forepaw offers a unique occasion to conduct a combined study of cutaneous fiber physiology and the putative structural morphology of the underlying receptor. The extremely sparse presence of Merkel complexes and Meissner corpuscles make a combined electrophysiological and histological study more feasible than previous studies where the receptor density rendered the correlation between receptor structure and function less certain. In addition, newer immunochemical staining methods have improved the ability to identify the well-known skin receptors with certainty. Taken together, these features offer an exceptional opportunity to clarify a long-standing ambiguity in somatosensory neurophysiology.

## Methods

### Surgical procedures

The experiments, conducted on adult Wistar rats weighing 250–500 g, were performed in accordance with guidelines from the Canadian Council on Animal Care and were approved by the animal ethics committee of the Université de Montréal. The rats were anesthetized with an initial 50 mg/kg intra-peritoneal dose of pentobarbital sodium and 0.05 mg/kg of buprenorphine. The anesthesia was maintained with supplementary doses for the duration of experimental recording, which was generally about 4 h.

The surgical exposure of the median nerve was accomplished by making a Z-like incision in the forelimb above the wrist. The connective tissue was incised and the muscles reflected to expose about 1.0 cm of the median nerve just above the carpal tunnel. The nerve was separated and raised from the surrounding tissue with a thin strip of latex inserted beneath the nerve. Throughout the recording period the nerve and surrounding tissue were kept moist with physiological saline. The supinated forepaw was fixed to a plexiglass plate and the extended digits were fixed with glue applied to the claws at the end of each digit.

### Microneurographic recording

The microneurographic recordings were conducted with glass-insulated, sharpened, tungsten electrodes with a resistance of 0.7–0.9 MΩ with an approximate tip exposure of one micron. Unitary action potentials were displayed on an oscilloscope and a window discriminator was used to send spike trains to a computer. The microelectrode was slowly introduced into the nerve at a proximal-to distal-angle of between 30° and 40°. Care was taken to ensure that the axon potentials were of sufficient amplitude to provide single unitary recordings.

### Receptive field testing

Once a single fiber had been adequately isolated the response to pressure with graded monofilaments was used to establish the threshold and extent of the receptive field. Monofilaments of increasing large caliber were tested up to 5× threshold. In early experiments calibrated Semmes Weinstein monofilaments (Bell-Krotosky 1987) were used to probe receptive fields, whereas in later recordings an IITC Electronic von Frey aesthesiometer^®^ provided an analog voltage proportional to the applied force, which could be recorded by a computer. In both cases the monofilaments were applied manually with only enough pression to produce flexion. Monofilament pressure was applied for 5 s and a minimum of 10 repetitions were recorded with a 5 s pause between applications. This procedure enabled the unequivocal identification of RA and SA fibers. In experiments with the monofilament equipped with a strain gauge, a force approximately 4× threshold was applied to the receptive field and a minimum of 20 replications were recorded on a laboratory computer.

### Histological staining procedures

At the conclusion of the electrophysiological study, the rats were sacrificed with a lethal dose of pentobarbital sodium and the glabrous skin of the forepaws was excised. The composition of 10 digits cut in 14 µ serial sections along the long axis of the digit and six pads from the palm was examined microscopically. The tissue was fixed in 4% paraformaldehyde in 0.1 M phosphate buffered saline (PBS) with a 7.4 pH for 5–12 h at 4 °C. After careful rinsing with 0.1 M PBS the tissue samples were kept for at least 24 h in 0.1 M PBS with 0.1% sodium azide and 30% sucrose for cryoprotection. The digit tissue was then flattened and frozen whereas the thenar pads were frozen without flattening. Subsequently 14 µ frozen sections were cut perpendicular to the skin surface along the longitudinal axis of the digit and prepared for antibody staining. Neural structures were identified in the glabrous skin using the combined antibodies PGP 9.6 and NF-200 as suggested by previous studies (Rice and Rasmusson 2000; Paré et al. 2002; Guinard et al. 1998; Le Master et al. 1999). Anti NF-200 selectively stains myelinated fibers as well as the Meissner corpuscles. We used PGP 9.6 to stain both myelinated and unmyelinated fibers and the Merkel cells. The antibodies were diluted in 1% bovine serum albumen (BSA) and 0.3% Triton X 100 in PBS. The slides were pre-incubated for 30 min. in PBS/BSA/Triton solution followed by exposure to anti-neurofilament NR-200 kDa (1:800 dilution, Chemicon International, Inc.) for 24 h in a humid atmosphere at 4 °C. The sections were then rinsed with PBS and re-incubated for 2 h in Cy3 (1:500, donkey anti-rabbit). After a second 30 min rinsing in PBS a second incubation was conducted with anti-PGP 9.5 (PGP 9.5 dilution 1:800 Ultraclone Ltd. UK) for 24 h at 4 °C. This was followed by a further incubation with Cy2 (1:2500). The sections were then washed and mounted on slides with 90% glycerin/10% PBS and cover slipped.

The sections were examined with a Nikon Eclipse microscope equipped for conventional fluorescence and tissue images were photographed with a Nikon digital camera. The global structure of the glabrous skin and the location and structure of the dermal–epidermal junction were visualized at 200×. At this magnification the border delimiting the dermis and epidermis was easily distinguished. A more detailed analysis of the Meissner corpuscles and Merkel cell was conducted at 400×. Finally, a three-dimensional tissue reconstruction of the terminal was accomplished using NeuroLucida^®^ software on a microscope equipped with a motorized stage in *X*, *Y* and *Z*-axes.

## Results

### Macroscopic description of the forepaw glabrous skin

**Fig. 1:**
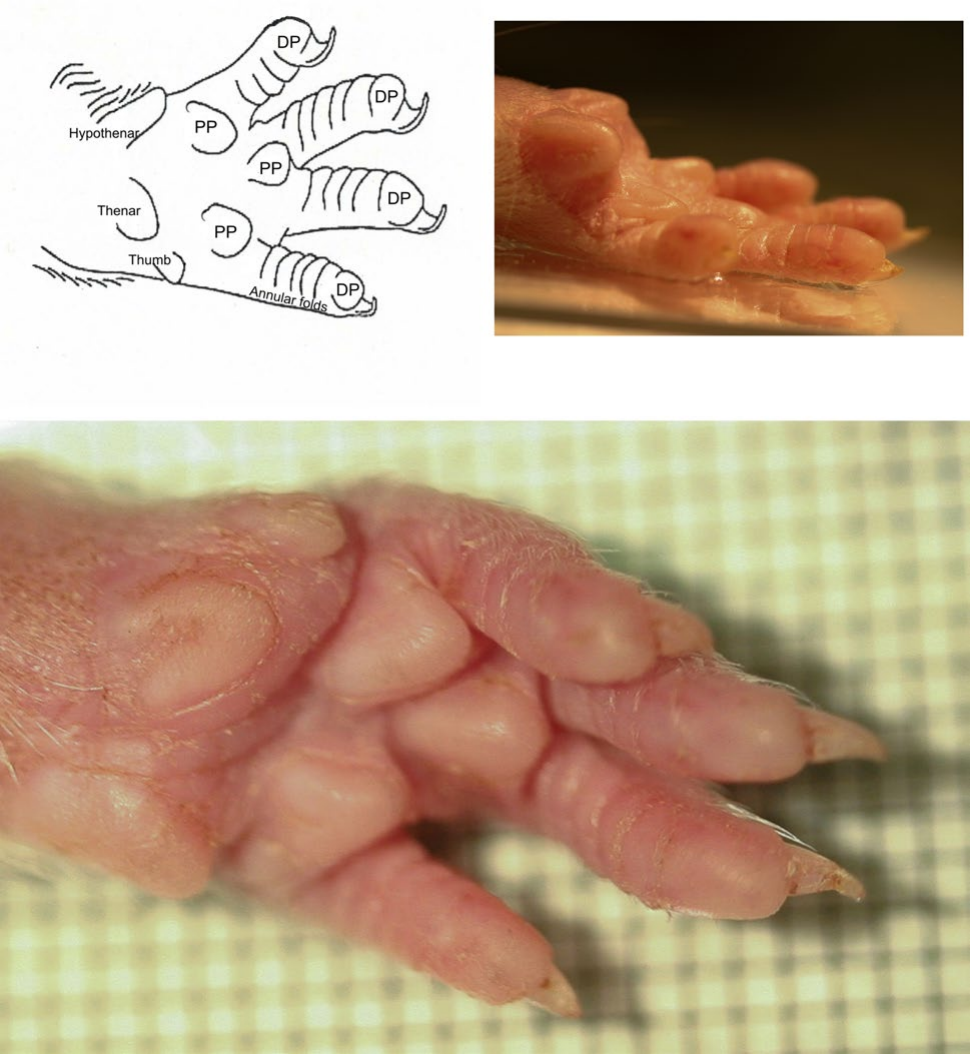
**A** *AF* annular folds, *DP* digital pad, *PP* palmer pads, *T* vestigial thumb. **B** Photomicrograph of digital pad. **C** Photomicrograph of digit pad (**a**), lateral border (**b**) and annular folds (**c**)

Despite a general resemblance of the rat forepaw to the human hand (Kimura et al. 2002), the absence of a functional thumb, the presence of sharply pointed pyramidal-like pads on the palm and the absent fingerprints on the digit extremities constitute some significant differences (see Fig. 1). The forelimb palm contains 5 very distinct pyramidal shaped pads; one each on the thenar and hypothenar eminences and three more arranged over the metacarpal bones. The glabrous skin over the distal phalanges differs from the more proximal phalanges by the presence of a digital pad that resembles the palmer pads and comprises about half the 7 mm total length of the digit. The thumb is rudimentary to the point of being almost absent. Similar to the cat paw pad the surface of the digital pad is smooth without fingerprint ridges and grooves (Bolanowski and Pawson 2003). The palm contains 5 protuberances 2 of which correspond to the thenar and hypothenar eminences. The 3 more distal palmer pads protrude 1.3 mm perpendicular to the palm from a 2.00 mm base and are arranged radially over the metacarpal joints. The digits are composed of three phalangeal bones. The distal digit is covered with a pad similar to the palmer pads but less pointed. In contrast to the palmer pad, the distal pad is shaped like a ridge running longitudinally, peaked in the middle and sloping away laterally on either side. When contacting a supporting surface, the claw covering the distal pad probably exerts a maximal strain on the skin covering the distal phalange. On the more proximal digit, the skin covering the first and second interphalangeal joints contains annular folds some of which correspond to the interphalangeal joints in addition to several additional folds that do not.

### Microscopic description of forelimb glabrous skin

**Fig. 2:**
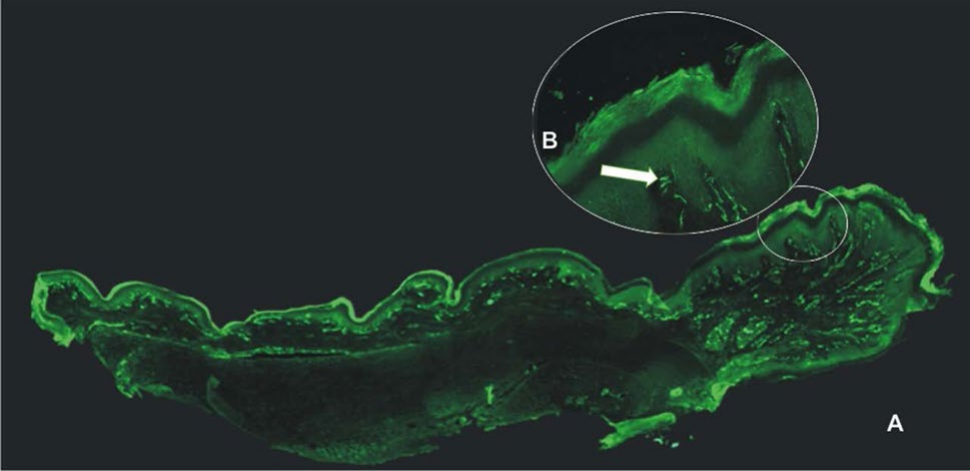
Photomicrograph montage of 14-mm sections of rat digit stained with anti-PGP cut perpendicular to the skin surface in the long axis. Photo taken with ×10 objective. **a** The section runs through the middle of the digit. The majority of receptors and papillae were located at the digit tip. **b** ×20 image of the same cut showing Meissner corpuscle at the apex of the digital papilla

In general, each digit was examined with about 90 sections cut longitudinally in 14 µm sections and comprising about 60 sections for each palmer pad. It was immediately apparent there were striking morphological differences both between the middle and the lateral distal digit and also between the middle distal digit and the annular skin covering the more proximal phalanges. We examined microscopically the composition of ten digits pads cut in 14 µ serial sections along the long axis of the digit and six pads from the palm. For the digits there was a striking morphological difference between the proximal digit skin and the middle of the distal volar phalange. The lateral distal regions near the hairy skin border and proximal areas were almost completely devoid of dermal papillae and the dermal–epidermal junction when present was irregular and indistinct compared to the central region beneath the pyramidal ridge. Within the central ridge of the distal pad the dermal papillae were distinct and regular in both shape and dimension (see Fig. 2). Unlike the primate digit there is no correspondence between the dermal papillae and fingerprint ridges because the stratum corneum in the rat is entirely smooth. With respect to the palmer pads all the dermal papillae were situated exclusively at the pyramidal peak in contrast to the base whereas they were absent.

### Meissner corpuscles

**Fig. 3:**
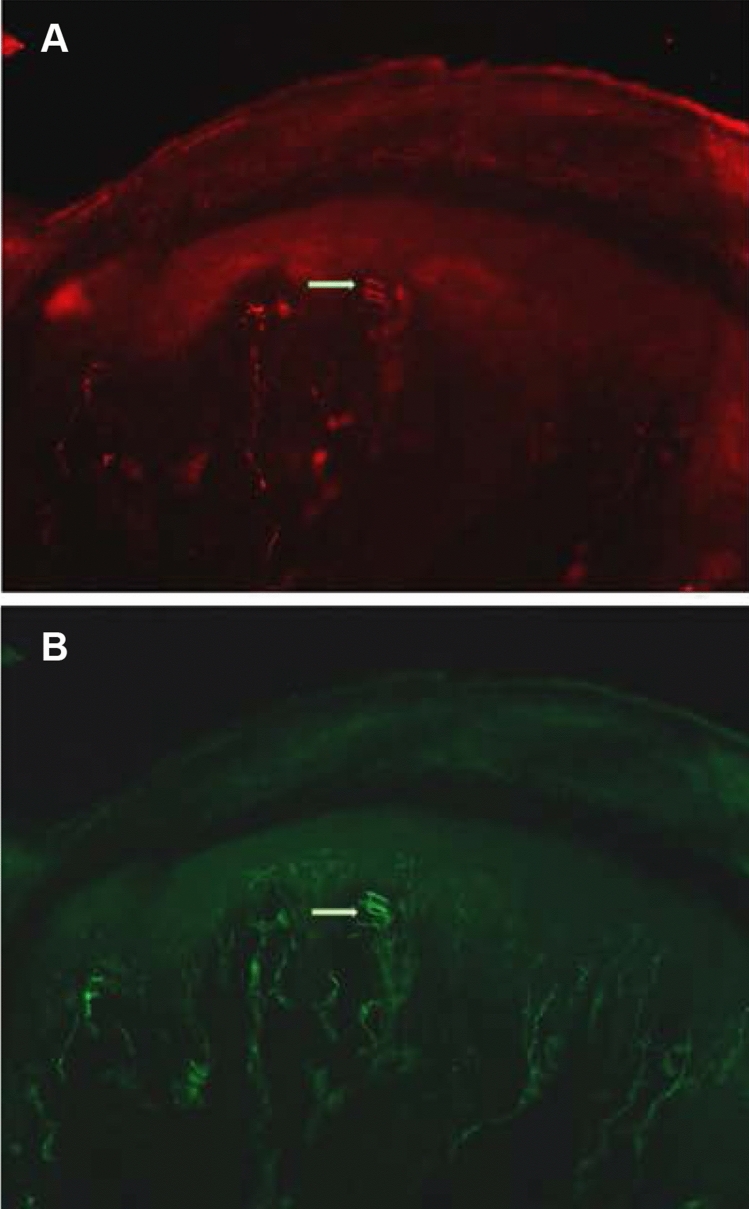
Anti-NF (**a**) and anti-PGP (**b**) immunofluorescence labelling of innervation in a 14 mm section of the distal volar pad of the rat digit, taken with the ×20 objective showing a Meissner corpuscle in a dermal papillae

Anatomical structures resembling the Meissner corpuscles (MC) from their size, location and innervation were found in the rat forepaw. They resembled previou**s** descriptions of Meissner endings in the human skin (Guinard et al. 1998; Castano et al. 1995; Nolano et al. 2003) and in other mammals (Bolanowski and Pawson 2003; Guclu et al. 2003; Paré et al. 2001, 2002). Consistent with earlier studies, the Meissner corpuscles were ovoid structures located the within dermal papillae with an average diameter of 30 µm ± 2 μm and an average length of 40 µm. Most frequently the same Meissner ending could be seen in two serial sections and less frequently in three consecutive sections. The long axis was oriented toward the epidermis perpendicular to the skin surface. As shown in Figs. 2 and 3, the majority of these receptors were located at the apex of the papillae without penetrating into the epidermis as described in the monkey (Bolanowski and Pawson 2003); Guclu et al. 2003; Paré et al. 2002). Both myelinated and unmyelinated fibers with and without varicosities could be seen entering the base of the corpuscle as shown in Figs. 2 and 3. The ovoid structures appeared to be encircled by 2 or 3 narrowly spaced fibers immunoreactive for anti NF200 corresponding to the myelinated fibers surrounding the lamellated Schwann cell that forms the base of the Meissner corpuscle (Fig. 3).

The distribution of Meissner corpuscles was closely associated with the presence of the dermal–epidermal papilla. The lateral edge of the digit near the hairy skin was devoid of both papillae and Meissner corpuscles and they were also absent in the skin covering the proximal third of the digit. In contrast, almost every papilla in the pyramidal peaks of the palmer pads and central distal digit ridge contained a Meissner corpuscle. Both the apexes of the thenar pads and the digit ridges contained an average number of 35 Meissner corpuscles. Assuming an average digit ridge length of 2.0 mm and an average width of 1.3 mm we calculated a mean density of 13 MC/mm^2^ for the digit tip compared to less than 0.9/mm^2^ for the remainder of the digit.

### Merkel cells complexes

**Fig. 4:**
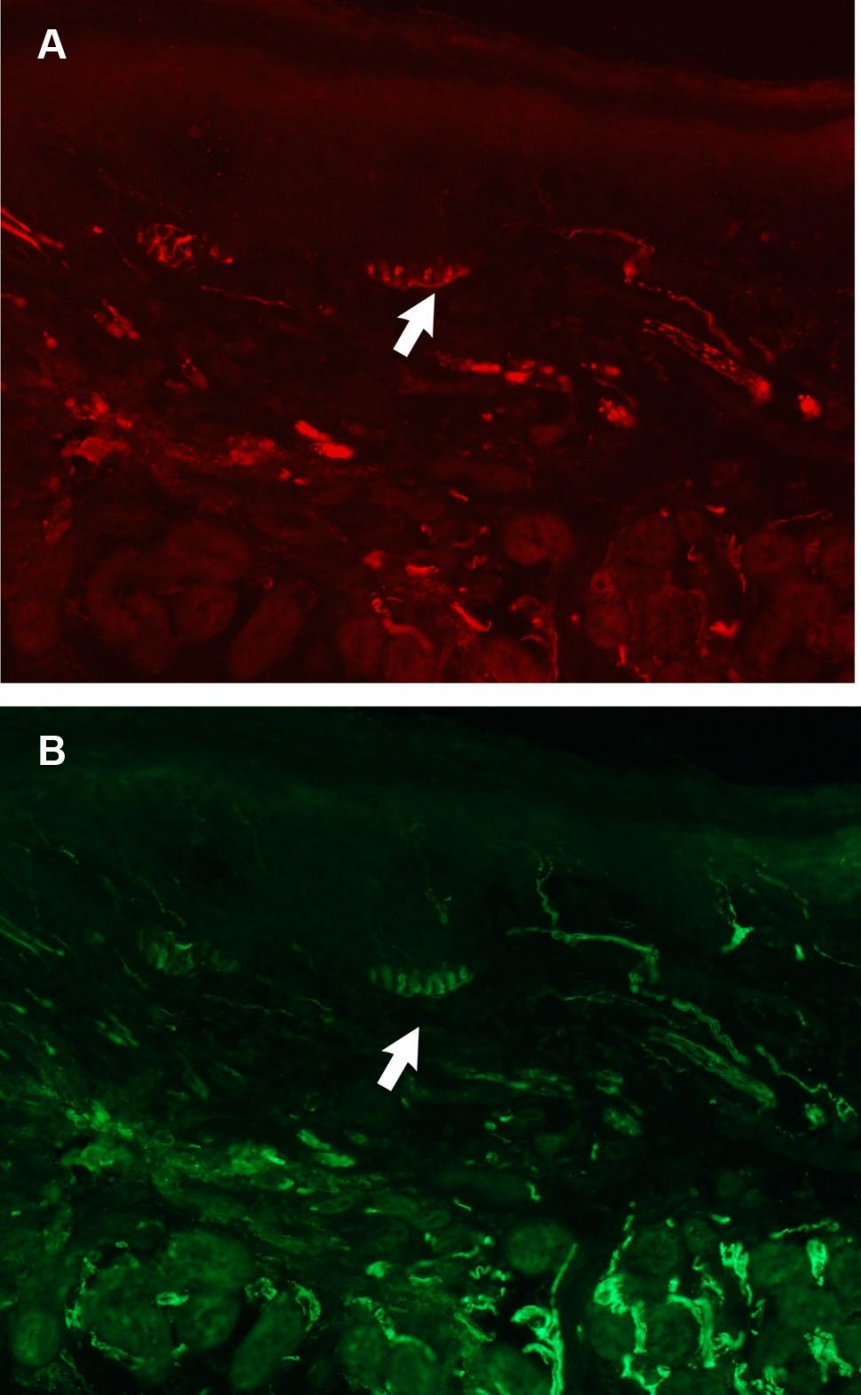
Anti-NF (**a**) and anti-PGP (**b**) immunofluorescence labelling of innervation in a 14 mm section of the distal volar pad of the rat digit, taken with the ×20 objective. Merkel cells (broad arrows) can be seen in the basal lamina of the epidermal ridges

The anti PGP9.5 was used to label Merkel ending complexes (MEC) as well as the afferents that innervate them. Similar to the glabrous skin of the primate hand (Paré et al. 2002), Merkel complexes in the rat were found in the dermal–epidermal border and most frequently at the deepest portion of the papillae. The ellipsoid Merkel cells were larger than the neighboring epidermal cells, and arranged in innervated chains of 5–8 cells approximately 50 μm in length (Fig. 4). The Merkel cell chains followed the contours at the base of the epidermal border with each cell equidistant from one another and linked together by a common axon. In most cases the stem axon formed “en passant” branches that contacted Merkel cells extending for 20 or 30 μ along the papillae border. Rarely the Merkel complexes could be seen to form more compact bouquets or clumps at the bottom of the papillae. Merkel cells located more superficially near the papillae apexes did not appear to be innervated. The total number of Merkel complexes in the distal digit pad was 11 on average, which is a density equivalent to about 4.2 MEC/mm^2^. The entire remainder of the digit was essentially devoid of Merkel complexes and their number rarely exceeded 4 yielding a low average density of 0.6 MEC/mm^2^. The average number of Merkel endings in the palmer pads was 21 and therefore considerably higher.

Although in most cases the Merkel cell complexes followed the contour of the papillae, they could occasionally be found immediately beneath the epidermis in areas without papillae such as was typically found at the lateral edge of the distal pad or in the palmer pads where both the dermal papillae and Meissner endings were extremely rare. The area without papillae seemed to contain only the chain endings rather than the more compact bouquet aggregates. Considering the very small size of the rat palmer pads, we did not attempt to calculate a density for Merkel complexes in the thenar pads to compare with the distal digit. Nevertheless, in both cases the Merkel complexes were concentrated near the peak of the distal digit ridge and at the summit of the palmer pyramid pads. However, the density of Merkel complexes is much greater in the palmer pads than at the digit extremity because the distal ridge is considerably larger than the palmer pad.

In summary, the palmer and digital pads have several similarities that distinguish these regions from the remainder of the glabrous skin. The distal phalangeal pads contained a mean density of 13 MC/mm^2^ and 4.2 MEC/mm^2^ compared to 0.9 MC/mm^2^ and 0.6 MEC/mm^2^ of the middle and proximal phalangeal regions. In our opinion the anatomical structure and localization of both the Meissner corpuscles and the Merkel cell complexes in the rat are virtually identical to those observed in the other mammals. These receptors were concentrated within a very limited region along the crest of the distal digital pad and the summit of the palmar pyramidal pads but the density and absolute number of receptors was considerable higher in palmar pads. The digital extremity and the palmer pads contained about the same number of Meissner corpuscles whereas there were approximately twice as many Merkel complexes in the palmer pads compared to the digit tips. The ratio of Meissner corpuscles to Merkel complexes was about 3.2/1.0 in the distal digit and 1.7/1.0 in the thenar pads.

### Electrophysiological recording

Single fibers were isolated in the median nerve just proximal to the carpal tunnel. Single units were fed into a window discriminator and recorded on a computer. From 139 partially tested fibers, 92 were held long enough for complete receptive field mapping and threshold analysis. Thirty-five fibers (38%) were identified as rapidly adapting (RA) whereas 57 (62%) were characterized as slowly adapting (SA) according to the conventional responses to dynamic and static pressure applied to the receptive field with monofilaments. All the recorded units had discrete and low threshold receptive fields. The absence of units with wide receptive fields sensitive to high frequency vibrations suggests that no Pacinian Corpuscles were included in our sample. RA receptors responded with a burst of activity to the dynamic application and withdrawal of light pressure to their receptive fields. The continued application of static pressure produced no activity. A small number (10/139) of receptors produced a burst of activity that outlasted the dynamic phase but the static phase activity was sporadic and unlike the majority of SA receptors and therefore they were excluded from the present study. Leem et al. (1993) also observed similar receptor activity in single fiber recordings from the sural nerve innervating the hind paw of the rat. Of the 57 SA recorded receptors 47 (86%) could be described as moderately slowly adapting (MSA) and 8 (14%) could be characterized as very slowly adapting (VSA) according to the classification criteria proposed by Pubols and Pubols (1973). Both types had a continuous discharge throughout the period of sustained pressure although the VSA discharge frequency was higher and more regular that the MSA units.

### Receptive fields

**Fig. 5:**
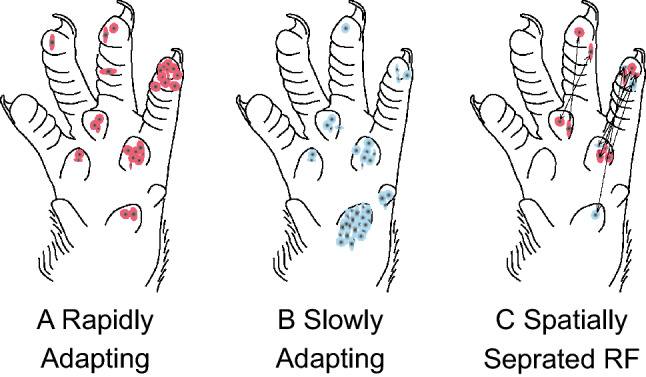
Shows the location of the recorded rapidly (**a**) and slowly adapting (**b**) receptors. Unit with spatially separated receptive fields are in **c**

Mapping the receptive fields (RF) was conducted with calibrated monofilaments at approximately 2× threshold pressure. Mapping started at the most sensitive part of the RF and probing radiated outward until no further responses were observed. The RFs of RA and SA were very similar in size and shape. Both were either round or oval with well delimited borders. The mean surface area for RA units was 2.0 mm^2^ ± 0.1 and 1.5 mm^2^ ± 0.1 although this was not statistically significant (*t* test *p* = 0.12). A total of 14 RA units and 4 SA units had RFs on the distal pads of the digits whereas 16 RA and 49 SA units had RFs on the palmer pad (see Fig. 5). In total there were approximately 3 times as many RA units compared to SA units at the digit extremity and 3 times as many SA units compared to RA units on the palmer pads. Figure 5 also illustrates 5 RAs and 2 SAs that had two discrete and widely separated receptive fields with one located in the palmer pad and the other on the digit extremity. In our opinion these responses reflect a common stem axon branching to innervate two distinct receptor sites.

### Thresholds

Thresholds were measured with calibrated monofilaments at the most sensitive part of the RF and the criterion was a 50% response with the most flexible monofilament (Janig 1971; Cain et al. 2001). The mean threshold pressure for RA units was 1.86 g ± 0.5 and 1.53 g ± 0.4 for SA units although this difference was not statistically significant (*t* test *p* = 0.20). However, a significant difference was noted between the receptor thresholds on the palm compared to the digit extremity. The mean threshold on the digit extremity was 5.19 g compared to 1.26 g on the palm and this was statistically significant (t-test p < 0.01). In our opinion this could be explained by either a larger number of receptors connected to a common stem axon on the palmer pads or a thicker or stiffer epithelium on the digit extremity.

### Discharge characteristics

**Fig. 6:**
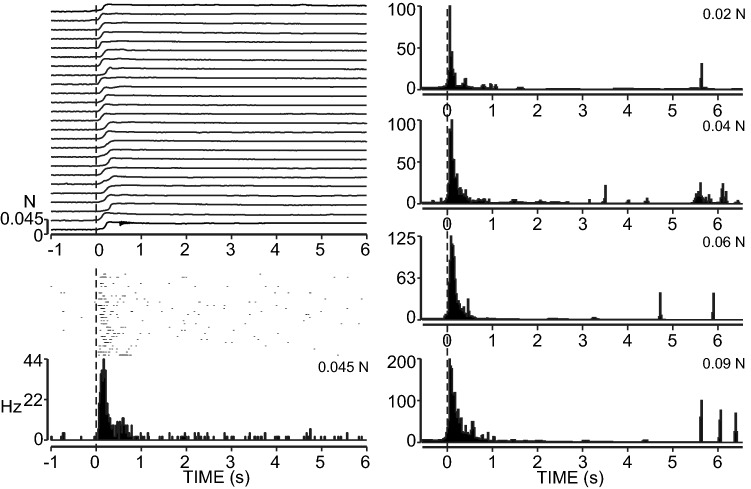
Repeated stimulation of a rapidly adapting unit at approximately ×4 threshold is shown on the left. Post-stimulus time histograms at right show the responses to graded applied forces ranging from 0.02 to 0.09 N

**Fig. 7:**
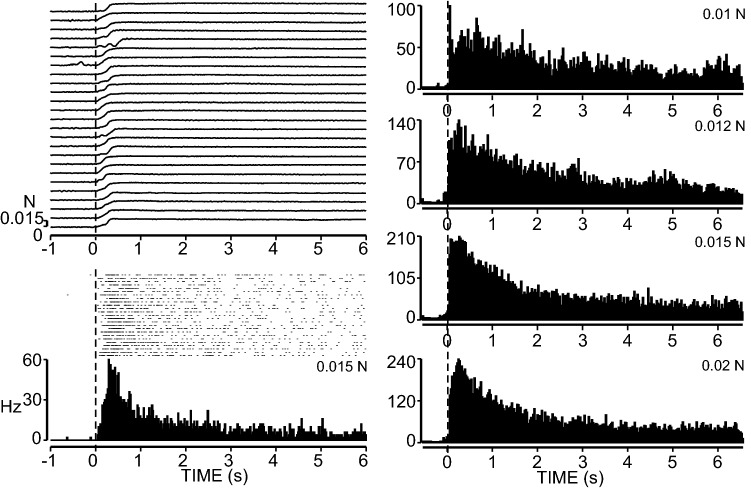
Repeated stimulation of a very slowly adapting (VSA) unit at approximately ×4 threshold is shown on the left. The stimulus application signal is noisy because of the high gain and very light force application. Post-stimulus time histograms at right show the responses to graded applied forces ranging from 0.01 to 0.02 N. Note the histograms are not to the same scale

**Fig. 8:**
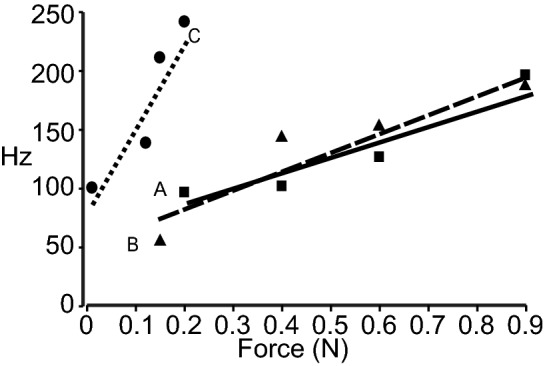
The mean responses to graded forces are shown for a RA receptor (**a**) and moderately slowing adapting receptor (**b**). Both receptor types show moderate activity increases in response to graded force applications. **c** A very slowly adapting receptor with a steep activity increase to much lower range of forces

The discharge properties of the receptor units were studied using subthreshold level monofilaments and increasing the stiffness to approximately 5× threshold. A typical RA discharge pattern is illustrated in Fig. 6. For the majority of RA and MSA receptors increasing the force applied with the monofilament had only a moderate effect on the peak discharge rate. However, in addition to their very low threshold the VSA units rapidly increased their discharge with the increased pressure applied to the RFs, reaching a plateau frequency at 0.02 N shown in Fig. 7. Figure 8 illustrates the mean peak discharge for the RA, MSA and VSA classes of receptor for a range of applied forces. Clearly the VSA units not only had the lowest thresholds but they also reached their plateau discharge rate at very low force levels at about 0.25 N.

To summarize, the size and shape of both RA and SA receptors was very similar. However, about 3.5 times as many RA units were recorded on the digital pads compared to SA units. Conversely there was a much higher concentration of SA receptors compared to RA receptors in the palmer pads. The digital pads are much more sparsely innervated than the palmer pads and the receptor thresholds were also much lower on the palmer pads. Although both RA and MSA receptors responded moderately to graded pressures applied with monofilaments, the VSA units had significantly lower thresholds and graded pressure responses at a much lower force threshold.

## Discussion

The principal objective of the present study was to compare the electrophysiological characteristics of single fibers recorded in the median nerve with a histological analysis of cutaneous receptors in the glabrous skin of the rat forepaw. The morphological descriptions as well as the dermal localization of both the Meissner Corpuscles and Merkel cell complexes were in agreement with previous accounts. (Botchkarev et al. 1999; Castano et al. 1995; Cauna 1956; Lumpkin et al. 2003; Munger et al. 1971; Rumio et al. 1995; Takahashi-Iwanaga and Shimoda 2003; Yamashita et al. 1993). Takahashi-Iwanaga and Shimoda (2003) described the Meissner corpuscles of the monkey as a stack of disks composed of axon terminals interlaced with the lamella of Schwann cells. Our observations agree with this description in which each corpuscle was encapsulated in a sheath of conjunctive tissue and connected by collagen fibers to the epidermal basal membrane. Our observations are also in agreement with Nolano et al. (2003)’s description of the human finger and Paré et al. (2002)’s depiction of the glabrous skin of the monkey hand. The Meissner corpuscles were invariably found at the apex of the epidermal papillae whereas the Merkel cells were most often located at the base of the folds. Using the same histological procedures, we found the shape and overall dimensions of the Meissner corpuscles to be identical to what Paré et al. (2002) obtained for the monkey finger. However, Meissner corpuscles appeared to be somewhat smaller than those described by Guinard et al. (2000) for the human.

### Meissner corpuscles

The histological preparation of the rat digits required flattening the tissue, which was not the case for the palmer pads and for this reason we are unable to compare the density of receptors between the palmer pads and the digit extremity with absolute certainty. However, we were able to document the regional distribution of Meissner corpuscles within the digit. The density of Meissner corpuscles in the distal digital pad of the rat was considerably less (13 MC/mm^2^) than either the monkey fingertip which has been variably estimated as 40.3 MC/mm^2^ (Bolanowski and Pawson 2003), or 60.4 MC/mm2 (Paré et al. 2002) or 33.0 MC/mm^2^ for the human (Nolano et al. 2003).

Using a mathematical algorithm and cholinesterase staining, Guclu et al. (2003) estimated the density of Meissner corpuscles in the cat, 3 species of monkey (baboon, owl monkey and Rhesus) and humans. For the baboon the 5.7 MC/mm^2^ density was even lower than what Iggo and Ogawa (1977) estimated for the cat (11.5 MC/mm^2^). Estimates for the human 17.3 MC/mm^2^ also seem remarkably low compared to the owl monkey and rhesus monkey at 41.7 MC/mm^2^ and 27.0 MC/mm^2^, respectively. Guclu et al. 2003 also failed to find any difference Meissner density between samples taken from the digits or the thenar skin. It seems likely that some of the density differences were due to differing staining methods and the mathematical estimation procedure as well as significant differences between species.

### Merkel cell complexes

Despite the controversies over whether Merkel cells are excitable and what their role in tactile discrimination might be (Fagan and Cahusac 2001; Haeberle et al. 2004; Johnson 2001; Lumpkin et al. 2003), there is widespread agreement about their morphological description. Apart from birds where the Merkel cells are located directly in the dermis (Saxod 1978), most investigators, including the present study, find that Merkel cell complexes are invariably located in the deepest layer of the epidermis, most frequently in the basal dermal papillae and fixed to the dermal membrane as described by Guinard et al. (1998) and Paré et al. (2002). Since not all Merkel cells are innervated, the greatest challenge was the identification of Merkel cells among the tangle of blood vessels and myelinated nerve fibers at the dermal–epidermal junction as well as the many unmyelinated free nerve endings stained with anti PGP9.5. The chain endings found in the rat closely resembled the description of Merkel rete papillae in the racoon (Munger et al. 1971) and the Merkel cell agglomerations in humans (Guinard et al. 1998). The number of Merkel cells contacted by a common axon was 24–36 in the racoon whereas in the human the number was thought by Guinard et al. (1998) to be considerably smaller and comparable to the rat (5–8 cells). Nevertheless, Paré et al. (2002) cautioned that it is often hard to tell the extent to which a single axon could provide branches to multiple Merkel cell complexes. In general, the dimensions of the Merkel cell complex in the rat (50 μ) were comparable to the human (80–100 μ, Guinard et al. 1998). Paré et al. (2002) suggested that Merkel cell complexes are of two types: bouquets or clumps and chains with a ratio of about 4:1 in the monkey. In agreement with Pare et al. (2002), we also noted two distinct spatial arrangements of Merkel cell complexes. However, in contrast to the monkey the majority Merkel cell complexes in the rat consisted of chains of cells connected to a common stem axon with only a small minority ending in tightly grouped bouquets. Although we did not observe the very long Merkel cell innervation chains up to 500μ in length described by Nolano et al. (2003) and Paré et al. (2002), the chain-like endings were more common than the clumped aggregates in the rat. Paré et al. (2002), also suggested that about 75% of the dermal–epidermal junction of the glabrous skin was occupied by Merkel cell complexes, which is in contrast to our observation of the rat digit where the complexes were very sparse except for the ridge at the digit tips and the peaks of the palmar pyramidal pads. At these locations the density of Merkel cells complexes was about 4.7 MEC/mm^2^, which is comparable to 4.0 MEC/mm^2^ found in the human fingertip by Nolano et al. (2003).

Our examination of the rat forepaw indicated that there were twice as many Merkel cell complexes in the palmer pad compared to the digit tip. This contrasts with Munger’s observation (Munger et al. 1971) on the racoon and many studies of other species showing a much higher concentration of Merkel cell complexes at the digit tip than in the palm. The ratio of Meissner corpuscles to Merkel complexes was 3.2 MC/mm^2^ to 1.0 MEC/mm^2^ in the rat digit tip and somewhat lower ratio at the peak of the palmer pads (1.7 MC/mm for every MEC). In contrast, Paré et al.(2002) reported almost 4 times as many Merkel cell complexes compared to Meissner corpuscles in the monkey hand.

### Electrophysiological results

Our data included one type of rapidly adapting receptor and 2 types of slowly adapting receptors. We found no units with spontaneous activity which might have corresponded to SAII receptor activity described by many investigators (Chambers et al. 1972; Goodwin et al.^1997^; Knibestol and Vallbo 1970; Vallbo and Johansson 1984; Vallbo et al. 1995). In the present study, two types of slowly adapting fibers fit the description of MSA and VSA discharge patterns described by Pubols and Pubols (1973) and Leem et al. (1993). Leem et al. (1993) found that VSA afferents were located in close proximity to joints in the hairy as well as glabrous skin and their sensitivity to joint motion suggesting a role in proprioception. Since our study did not examine either the hairy skin or joint movement, we are unable to comment on this aspect. Several observations in the present study were previously noted by Leem et al. (1993) including the greater occurrence of RA receptors in the digit tip ridges and the concentration of large fiber receptors in the palmer pad and at the digit extremity compared to the remainder of the glabrous skin.

The results of the present study indicate a RA/SA ratio of 3.3/1.0 on the digit tips compared to the inverse ratio of 1.0/3.0 for the palmer pads. That is, there is a prevalence of rapidly adapting receptors on the digit tips and the converse for the thenar pads. However, numerically overall there were more slowly adapting receptors (62% SA vs 38% RA) in the forelimb glabrous skin of the rat. Studies in other species appear somewhat contradictory. Coleman et al. (2001) found a 55% SA vs 45% RA receptor ratio in recordings from the median and ulnar nerves of the marmoset. They also found a greater number of receptive fields on the palm (60%) compared to the digits (40%). From a microneurographic analysis of the median nerve in human subjects, Goodwin et al. (1997), and Knibestol and Vallbo (1970) all found a greater number of slowly adapting receptors. In contrast Johansson and Vallbo (1979) found 56% RA versus 44% SA receptors with receptive fields on the palm and fingers in humans and Talbot et al. (1968) reported 18% more rapidly adapting receptors in the median nerve of the monkey. Since all these authors employed essentially the same microneurographic techniques it might be supposed that the differences arise from the different nerve fascicles explored and the thenar or digit area explored for receptive fields.

An interesting comparison and contrast can be made between our findings in the rat forepaw and the much larger sample of receptors recorded by Johansson and Vallbo (1979) for the human hand. The most striking difference is the overall absence of low threshold receptors in the rat forepaw except for the palmer pads and digit tips. A second contrast is the apparent higher density of innervation of the palmer pads in the rat compared to the fingertips in the human. Despite the fact that SA fibers comprised the overall majority (62%) of receptors in our sample, it is striking that there were three times as many RA compared to SA receptors recorded with receptive fields on the distal digit tip. This would appear to agree with similar higher proportion of RA receptors at the digit extremities in both monkeys (Darian-Smith and Kenins 1980) and humans (Johansson and Vallbo 1983). Conversely, we found about three times as many SA receptors on the thenar pads compared to the digits. It would appear that to be useful a critical concentration or receptor density may be necessary to sustain a functional utility. In this regard one might speculate that for the rat, the digit extremities are more useful in detecting shear and slip whereas the palms are more useful for texture and shape derived from sustained contact during object manipulation.

### Correlation between morphology and physiology

One of the original objective of this study was to take advantage of the sparse innervation of the rat forepaw to establish a correlation between rapidly and slowly adapting characteristics and the underlying receptor morphology. The histological analysis clearly indicated a far greater density of both Meissner corpuscles (13 MC/mm^2^) and Merkel cell complexes (4.2 MEC/mm^2^) in the middle portion of the digit extremity compared to the more proximal phalanges, (0.9 MC/mm^2^). In addition, a greater proportion of RA to SA receptors (3.3/1.0) was recorded on the digit tips, which would seem to suggest that Meissner corpuscles are indeed the morphological substrate of rapidly adapting responses. Similarly, the opposite RA to SA ratio (1.0/3.0) for the palmer pads would appear to concord with a lower ratio of Merkel cell complexes (4.7 MEC/mm^2^) to Meissner corpuscles in the palmer pads (1.0/1.7). These data tend to support the general consensus that Merkel cell complexes are indeed the morphological substrate of slowly adapting responses. Nevertheless, our conclusions are probable inferences rather than an unequivocal proof. Paré et al. (2001, 2002), pointed out that Meissner corpuscles have at least two distinctly different unmyelinated innervations the function of which is as yet to be fully determined. In addition, Paré et al. 2002), also noted that Merkel cell complexes have distinctly different branching patterns; clump and chain endings. The physiological correlates of these morphological features await further research.

## Data Availability

Available if requested.
